# A 28 GHz 2 × 2 Antenna Array with 10 Beams Using Passive SPDT Switch Beamforming Network

**DOI:** 10.3390/s21217138

**Published:** 2021-10-27

**Authors:** Firas Abdul Ghani, Amir Mohsen Ahmadi Najafabadi, Heba Saleh, Murat Kaya Yapici, Ibrahim Tekin

**Affiliations:** 1 Electronics Engineering, Sabanci University, Istanbul 34956, Turkey; firas@sabanciuniv.edu (F.A.G.); amirahmadi@sabanciuniv.edu (A.M.A.N.); hebasaleh@sabanciuniv.edu (H.S.); mkyapici@sabanciuniv.edu (M.K.Y.); 2 Sabanci University SUNUM Nanotechnology Research Center, Istanbul 34956, Turkey

**Keywords:** 5G antenna array, passive beamforming network, switched beams, SPDT switches

## Abstract

In this paper, a dual-polarized four-port 2 × 2 series fed antenna array operating at 28 GHz with beam-switching capability is proposed. The antenna array uses a simple passive beamforming network to switch the main beam. The presented antenna design is suitable for 5G user equipment and high data rates applications by which it has a compact size with low cost and complexity. The size of the antenna is 37.2 × 37.2 mm${}^{2}$ including the ground plane, and it produces 10 different switched beams by using only two simple 3 dB/90${}^{\circ }$ couplers which create the required amplitudes and phase excitations for the antenna elements. A one-port simple feeding mechanism including Peregrine PE42525 SPDT switch modules and a power divider is used to generate and measure the 10 switched beams. The antenna design is implemented on a two-layer 0.203 mm thick low-loss (tanδ = 0.0027) Rogers 4003C substrate, and it has a measured 10 dB impedance bandwidth of 4 GHz (14.3%, from 26 GHz to 30 GHz) for all ports. Measured peak isolation between any dual-polarized ports of the antenna is better than 30 dB. The antenna has an average measured realized gain of 8.9 dBi and around 10 dB side lobe level (SLL) for all beams. The antenna has 3-dB coverage of 80${}^{\circ }$ to 90${}^{\circ }$ in 2D space and it has a maximum of ±26${}^{\circ }$ beam-steering angle. The antenna is designed and simulated using Ansys HFSS and fabricated using regular PCB processing.

## 1. Introduction

5G (5th generation) mobile networks using millimeter-wave (mm-Wave) spectrum provide large bandwidths and multi-gigabit communication services [1]. Due to the complexity of the 5G communication system and different propagation characteristics compared to lower frequencies, researchers need a low-cost but high-gain and steerable antenna system with moderate performance to overcome the new challenges [2]. Antennas with beam-steering capability can significantly improve the reliability and performance of communication link through an improved signal to noise ratio (SNR) at the receiver without increasing the transmit power [3,4,5].

For mm-Wave applications, the multi-beam antenna arrays with steerable beams in two dimensions and dual-polarization are attractive candidates to achieve multispot coverage and high channel capacity [6,7,8,9,10,11,12]. One of the design complexities related to 2D multi-beam antenna arrays is high cross-polarized radiations and high integration complexity of the beamforming networks. The beamforming networks (BFN) are used to excite the radiating elements with the required phase and amplitudes to steer the beam in the intended direction. The BFNs can be obtained by either digital or analog circuits [13,14].

A variety of works and research have been reported to realize BFN to feed 1D and 2D beam-switching antenna arrays. Previously in [15], the 2 × 2 antenna array fed by two couplers has been proposed; however, only simulation results are provided. In [4,16], two-dimensional beam-scanning array antennas for 5G wireless communications are proposed, but simulation results are provided for only four beams for [16] and phase shifters are used to steer the beams in [4]. Insertion loss caused by phase shifters at 28 GHz can go up to 8 dB which will affect the signal sensitivity of the antenna system [17]. In [18,19], a lens-based beamformer (Rotman lens) is used to steer the beams of the proposed antennas. In [18], the antenna operates at 24 GHz and has two layers, and the Rotman lens feed the antenna through slots. In [19], a dual-polarized tapered slot-line antenna array that is fed by Rotman lens air-filled ridged-port is proposed. Both of the works have high complexity of design, cost, and loss. In [20], a 4 × 4 butler matrix is used to feed a 4 × 10 series fed antenna array operating at 5G 60 GHz frequency. The size of the butler matrix will be larger at 28 GHz, and it might not be suitable for the mobile phone application. In [21], a 4 × 8 butler matrix is used to feed a 8 × 10 antenna array operating at 38 GHz. The antenna uses three substrates and four ports to generate four switched beams. The works in [22,23,24] focused on the feed network by designing Substrate Integrated Waveguide (SIW) Butler Matrix and feeding components. In [25], a circularly polarized multi-beam antenna array based on magneto-electric (ME) dipole is proposed and is fed by SIW 5 × 6 Butler matrix which can generate five switched beams. Multibeam antennas realized using dielectric lenses and reflectors are simple, but they are bulky and expensive [26,27].

As seen from the previous works, many of the designs offer single-polarization radiation patterns, and those designs that use butler matrix usually use a large antenna array to compensate for the losses coming from those huge BFN. SIW feeding networks can provide wide bandwidths, but they are expensive and difficult to fabricate and implement. Most of the previous designs might not be suitable for 5G mobile phones that have cost and space limitations in antenna implementation.

In this work, a dual-polarized 2 × 2 series fed antenna array operating at 28 GHz is proposed. The array uses a simple passive beamforming network (two 3 dB/90${}^{\circ }$ couplers) as the main controllers that create the required amplitudes and phase excitations for the antenna elements to generate 10 switched beams.

This paper is organized as follows. Section 2 shows the working principle of the proposed antenna system. In Section 3, fabricated antenna and measured S-parameters are shown. Section 4 shows the simulated and measured radiation patterns of the antenna and compares the work with other works mentioned in the literature. Section 5 concludes the paper.

## 2. Working Principle of the Proposed Antenna System

**Figure 1:**
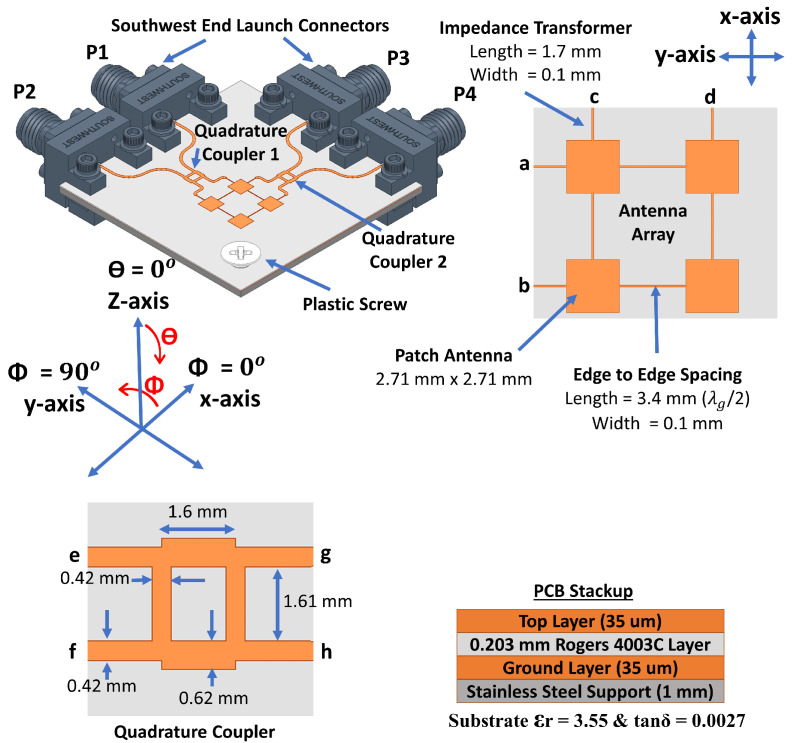
Proposed 2 × 2 antenna array with two couplers.

The optimized architecture of the proposed 2 × 2 series fed antenna array system is shown in Figure 1. The antenna array is designed to work at 28 GHz. The overall antenna size is 37.2 × 37.2 mm${}^{2}$. Each one of the four square shaped patch antennas (2.71 × 2.71 mm${}^{2}$) is radiating at 28 GHz and is fed from two ports through the impedance transformers. Those impedance transformers (L = 1.7 mm and W = 0.1 mm) are used to match the edge impedance of a single patch (400 ohms at 28 GHz) to 50 ohms at ports a, b, c, and d. Each of the edge-to-edge spacing transmission lines has a length of 3.4 mm and a width of 0.1 mm. Spacing between patch edges is designed to be equivalent to $\lambda_{g}$/2 so that phase is preserved the same between patch edges, where $\lambda_{g}$ is the guided wavelength at 28 GHz for Rogers 4003C substrate. Ports a and b of the antenna array are connected to quadrature coupler 1 which is designed to operate at 28 GHz band. The other two ports of the antenna are connected to quadrature coupler 2. Those couplers are extended with longer transmission lines so that Southwest End Launch connectors can fit the design.

The antenna is implemented on a two-layer 0.203 mm thick low-loss (tanδ = 0.0027) Rogers 4003C substrate by which the top layer is used to implement the antenna with the beamforming network and the bottom layer is used for ground. The antenna design is supported by a 1 mm thick stainless-steel plate which is designed with the same size as the ground layer and drilled at the connectors’ screws positions. Steel support will provide strong mechanical stability while measuring the antenna, and it provides a good connection between the ground layer of the antenna and the ground of each connector. As shown, a 3 mm hole is drilled at the corner of the antenna system, and a plastic screw is placed to support the antenna–steel layer connections at that corner. Other areas of the steel layer are tightly connected to the ground layer of the antenna through the grounding and clamping plates of the connectors that are fixed using connectors’ screws. Design and optimization of the proposed antenna system are done using Ansys HFSS 2020R2 version.

**Figure 2:**
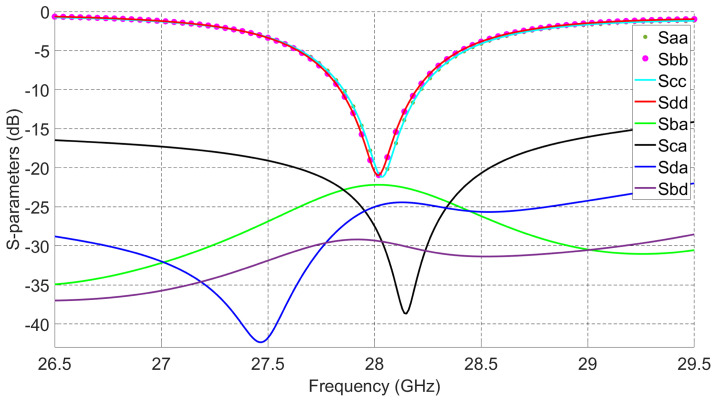
S-parameters of the 2 × 2 series fed patch antenna array.

For the 2 × 2 series fed antenna array, each radiating patch is fed from its edges’ centers through two orthogonal feeds (impedance transformers). This feeding mechanism provides high interport isolations between ports a, b, c, and d at the intended operating frequency. The reason behind that is the polarization diversity; in other words, when the antenna is fed through ports a and b, the polarization will be across ϕ = 90${}^{\circ }$, and when fed through ports c and d, the polarization will be across ϕ = 0${}^{\circ }$. This orthogonality between both polarizations provides high isolation between orthogonal ports. For example, as shown in Figure 2, isolation between ports a and c is better than 15 dB from 26.5 GHz to 29.5 GHz and reaches around 40 dB at 28.1 GHz. The other ports have isolations better than 20 dB for the whole targeted frequency band. The 2 × 2 antenna array design can reach 360 MHz 10 dB impedance bandwidth and 650 MHz 6 dB impedance bandwidth, which can be used for mobile phone applications [28]. Such dual-polarized 2 × 2 series fed patch antenna array with high interport isolations provides 2D symmetric beam-scanning and steering capabilities with Low SLL [4].

The 2 × 2 series fed antenna array has been first designed and optimized to have maximum simulated realized gain at one polarization (θ = 0${}^{\circ }$, ϕ = 0${}^{\circ }$ polarization) when the antenna is fed from ports c and d simultaneously, and another maximum at the second polarization (θ = 0${}^{\circ }$, ϕ = 90${}^{\circ }$ polarization) when fed from ports a and b simultaneously. By using different excitation combinations of in-phase two ports simultaneous excitation and 90${}^{\circ }$ out of phase two ports simultaneous excitations, 10 different switched beams can be obtained from the 2 × 2 series fed antenna array, as shown in Figure 1.

**Figure 3:**
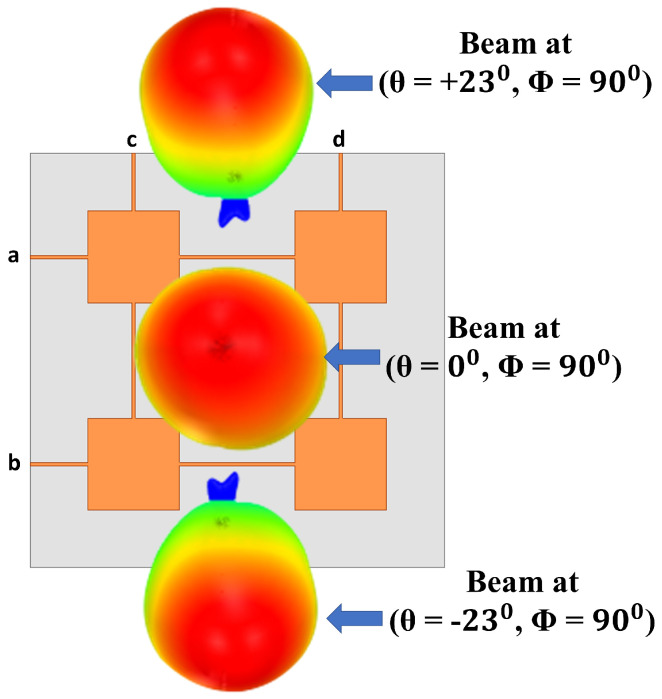
View of 3 switched beams excited using only ports a and b.

The theoretical beam positioning angles (θ and ϕ) for 2D phased antenna arrays in the spherical coordinate system are given by:

(1) $$\theta \;=\;\arcsin\left(\sqrt{{\left(\frac{\beta_{x}}{kd_{x}}\right)}^{2}+{\left(\frac{\beta_{y}}{kd_{y}}\right)}^{2}}\right),\;\phi \;=\;\arctan\left(\frac{\beta_{y}d_{x}}{\beta_{x}d_{y}}\right)$$

where $\beta_{x}$ and $\beta_{y}$ are the progressive phase differences between antenna array ports along with *x* and *y* directions, respectively. $d_{x}$ and $d_{y}$ are the inter-element spacing along with *x* and *y* directions, and in the current 2 × 2 series fed antenna array, they are 6.11 mm (≈0.57$\lambda_{0}$ at 28 GHz), and *k* is the wavenumber (2π/$\lambda_{0}$) in free space. When only ports a and b are excited while ports c and d are terminated with 50 ohms, according to Equation (1), the beam will be directed to (θ = +26${}^{\circ }$, ϕ = 90${}^{\circ }$) when the progressive phase difference ($\beta_{x}$) between ports a and b is +90${}^{\circ }$ and it will be directed to (θ = −26${}^{\circ }$, ϕ = 90${}^{\circ }$) when $\beta_{x}$ = −90${}^{\circ }$. When $\beta_{x}$ = 0${}^{\circ }$, the beam will be directed to (θ = 0${}^{\circ }$, ϕ = 90${}^{\circ }$ polarization). The 3D view of those three beams is shown in Figure 3. The 90${}^{\circ }$ progressive phase shift can be achieved by using quadrature couplers.

To obtain all phase and amplitude excitations needed to generate 10 switched beams in 2D space covering the four (4) quadrants, a very simple passive beamforming network using two symmetrical quadrature couplers is used. The proposed antenna system has good signal sensitivity because it does not include phase shifters to steer the beams which cause losses for beam-steering systems at mm-Wave frequencies. Insertion loss caused by phase shifters at 28 GHz can go up to 8 dB [17].

One coupler is connected to each side of the 2 × 2 series fed antenna array as shown in Figure 1. The optimized design of the coupler operating within the 28 GHz band is also shown in Figure 1. When feeding the coupler at port e, the power is divided equally between ports g and h with 90${}^{\circ }$ phase shift. Feeding at port f will give the same power outputs but with an opposite phase difference. Feeding ports e and f simultaneously will divide the power equally between ports g and h with the same phase. Table 1 shows ten (10) different feeding combinations for the couplers’ inputs to generate 10 different switched beams from the 2 × 2 series fed antenna array. The ON term refers to port excitation and the OFF term refers to 50-ohm termination. The first three beams are generated using ports 1 and 2 and the next three beams are generated by using the second side of the antenna system (ports 3 and 4). The last four beams are generated by using one port from each side of the antenna system. Out of ten (10) beams, four (4) beams (1, 3, 4, and 6) are generated using only a single port excitation, and the rest of the beams are generated by using simultaneous in-phase two-port excitations. Simulated and measured S-parameters of the antenna together with radiation patterns of the switched beams are discussed in the next sections.

## 3. Measured S-Parameters

**Figure 4:**
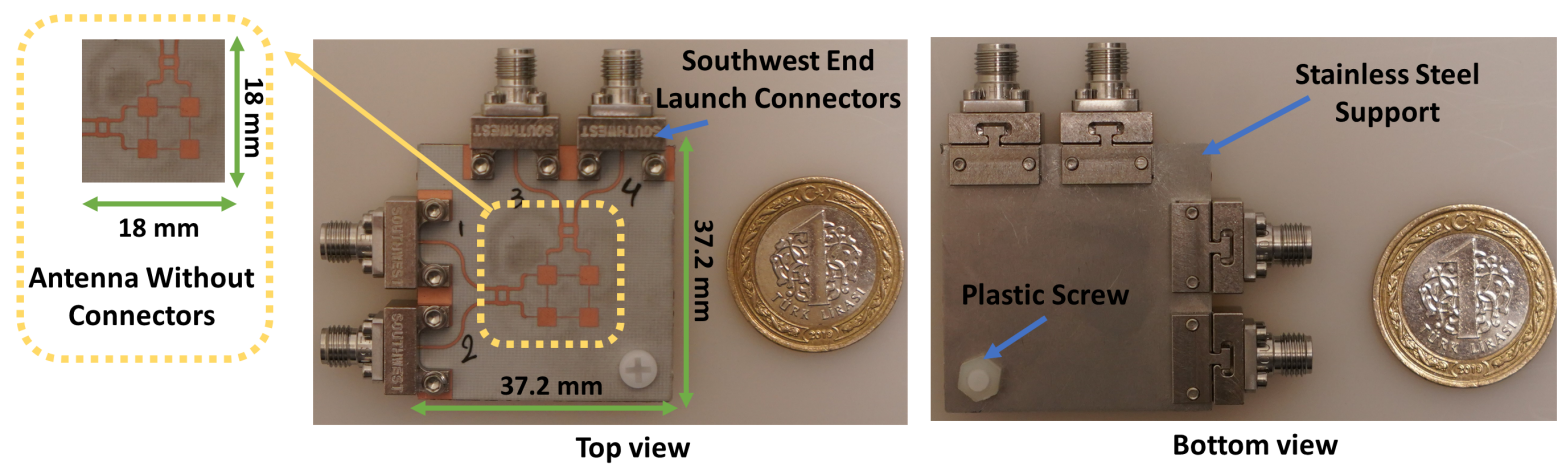
Top and bottom view of the fabricated antenna array.

**Figure 5:**
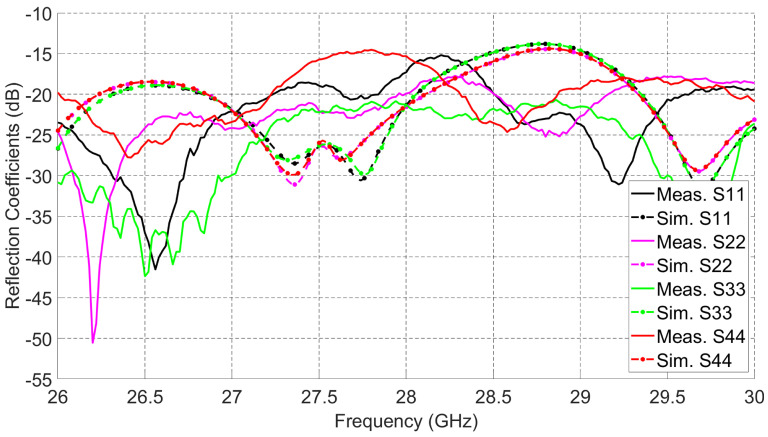
Measured and simulated reflection coefficients.

The fabricated prototype of the proposed multi-beam antenna system is given in Figure 4. It has a compact size (18 × 18 mm${}^{2}$) that suits regular 5G mobile devices. The antenna is tightly fixed with the connectors by using stainless steel support. A 50 GHz network analyzer Agilent PNA 5245A is used to perform the S-parameters measurements. Figure 5 presents the simulated and measured reflection coefficients ($S_{11}$, $S_{22}$, $S_{33}$, and $S_{44}$) at ports 1, 2, 3, and 4, respectively. The simulated and measured results show reasonable agreement. The measured 10 dB impedance bandwidth for return loss is 4 GHz (14.3%, from 26 GHz to 30 GHz).

**Figure 6:**
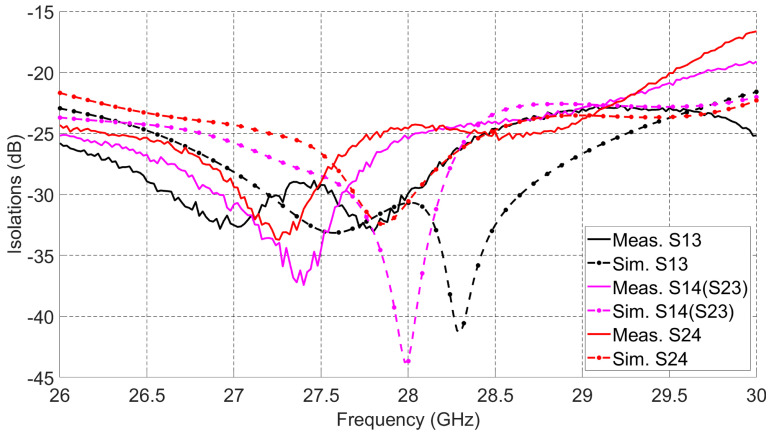
Measured and simulated isolations.

Quadrature couplers connected to the 2 × 2 series fed antenna array increase the 10 dB impedance bandwidth of the overall design when compared to the bandwidth of the 2 × 2 series fed antenna array alone. Those couplers have wide 10 dB impedance bandwidth at high frequencies. The measured and simulated isolations between antenna ports from different sides are given in Figure 6. The antenna array achieves better than 15 dB isolation from 26 GHz to 30 GHz and peak isolation better than 30 dB for any pair of dual-polarized ports. High interport isolation between the dual-polarized ports enables the designed array to achieve switching capabilities with high gain and low SLL.

## 4. Radiation Patterns of the Proposed Antenna System

### 4.1. Simulated Switched Beams

**Figure 7:**
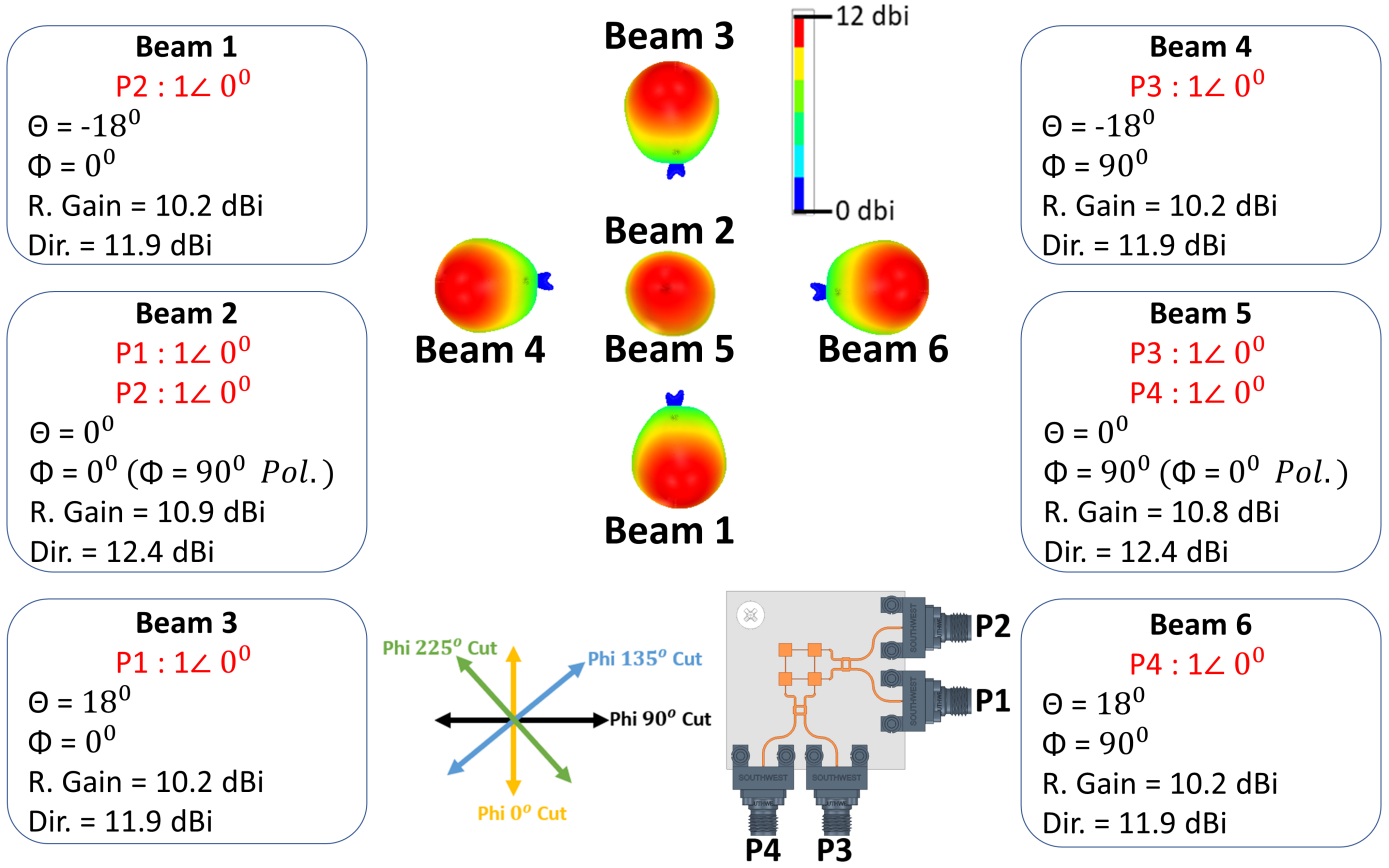
Six (6) simulated switched beams.

**Figure 8:**
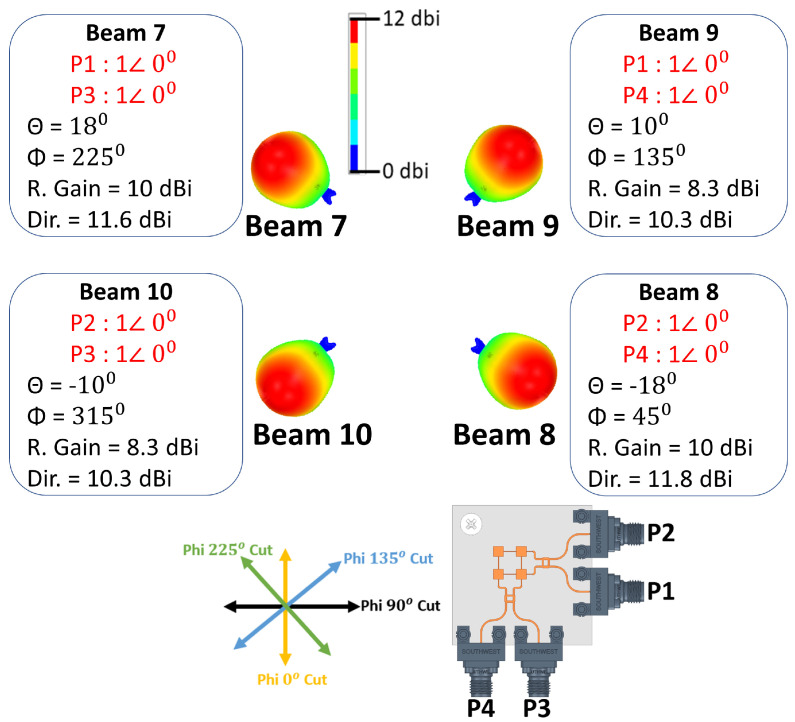
Four (4) simulated switched beams.

The antenna system shown in Figure 1 is simulated to verify the beam-switching capability of the 2 × 2 series fed antenna array connected to two quadrature couplers. The input ports of the two quadrature couplers are excited according to Table 1 to obtain ten (10) switched beams. For each simulated beam, directivity and maximum realized gain angle (θ and ϕ) are calculated and they are given in Figure 7 and Figure 8. The first six (6) beams shown in Figure 7 have an average realized gain of 10.4 dBi, and they are obtained by simulating ports from one side of the antenna system. Three (3) beams are obtained along with Phi 0${}^{\circ }$ cut when using the antenna side of ports 1 and 2 while terminating ports 3 and 4. The other three (3) beams can be obtained along with Phi 90${}^{\circ }$ cut when using the antenna side of ports 3 and 4 and terminating the other two ports. For example, exciting port 2 only and terminating each of the other three ports with 50 ohms loads generates beam 1 at (θ = −18${}^{\circ }$, ϕ = 0${}^{\circ }$) with a maximum realized gain of 10.2 dBi and directivity of 11.9 dBi. Exciting port 1 only will switch the beam to (θ = +18${}^{\circ }$, ϕ = 0${}^{\circ }$), which is beam 3 with a realized gain of 10.2 dBi, and when both ports 1 and 2 are excited simultaneously with in-phase and equal current magnitudes, beam 2 is obtained at (θ = 0${}^{\circ }$, ϕ = 0${}^{\circ }$) with a realized gain of 10.9 dBi. The polarization of beam 2 is across ϕ = 90${}^{\circ }$ cut. The other three beams (beams 4, 5, and 6) with similar θ angles across ϕ = 90${}^{\circ }$ cut and with similar realized gain values can be obtained when using the antenna side of ports 3 and 4 and terminating each of the other two ports with 50 ohms. Beams 2 and 5 have the same θ angle (θ = 0${}^{\circ }$), but they have different ϕ polarizations.

In-phase and equal current magnitude excitations of two ports simultaneously from both sides of the antenna system can generate four (4) switched beams as depicted in Figure 8. When ports 1 and 3 are excited at the same time, the antenna’s main beam switches to (θ = +18${}^{\circ }$, ϕ = 225${}^{\circ }$), which is beam 7 with 10 dBi realized gain, and when ports 2 and 4 are excited simultaneously, beam 8 at (θ = −18${}^{\circ }$, ϕ = 225${}^{\circ }$) is radiated with 10 dBi realized gain. The last two beams (9 and 10) are directed to (θ = ±10${}^{\circ }$, ϕ = 135${}^{\circ }$). Both beams have 8.3 dBi realized gain and they are excited using ports (1 and 4) for beam 9 and ports (2 and 3) for beam 10.

### 4.2. Feed Network for Radiation Patterns Measurements

**Figure 9:**
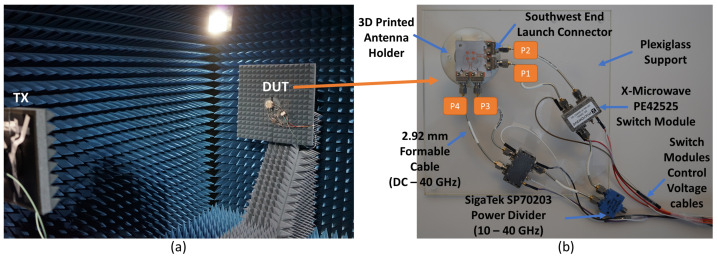
(**a**) Sabanci University Anechoic Chamber. (**b**) Antenna measurement components.

Radiation patterns of the 10 switched beams of the fabricated antenna shown in Figure 4 are measured and verified. Measurements are done at the Anechoic Chamber of Sabanci University which is shown in Figure 9a, and all measured data of the antenna under test are recorded including beam gains, side-lobe levels, beam position angles, and cross-polarization gain levels.

A simple measurement mechanism with a reduced number of controllers is used for measuring the ten (10) switched beams of the proposed antenna system. In Figure 9b, the antenna setup and the feeding network components are shown. To fix the antenna inside the Anechoic Chamber, an antenna holder was designed and printed using a 3D printer. In addition, a transparent plexiglass support was produced and fixed with the antenna holder to hold the feeding network components. SigaTek SP70203 power divider is used as the first component of the feeding network, and it operates from 10 GHz to 40 GHz, which covers the targeted 5G frequency band of the proposed antenna system. The outputs of the power divider are connected to two in-phase high frequency 2.92 mm 3-inch formable cables from Centric RF that operate up to 40 GHz. Two X-Microwave switch modules fed by the power divider are used to switch the coming signal between the antenna coupler ports. Each of the switch modules has an UltraCMOS SPDT RF switch (PE42525) from Peregrine Semiconductor. The switch chip operates from 9 kHz to 60 GHz, and at 50 GHz, it exhibits 1.9 dB insertion loss and 37 dB isolation which makes it a suitable choice for the proposed antenna measurements at 5G frequency bands [29]. Two in-phase 4-inch 2.92 formable cables are used to connect each switch module to antenna coupler ports (P1, P2, P3, and P4) as depicted in Figure 9b. The switching mechanism of those modules is controlled via voltage control cables by applying different voltage levels to each switch.

**Figure 10:**
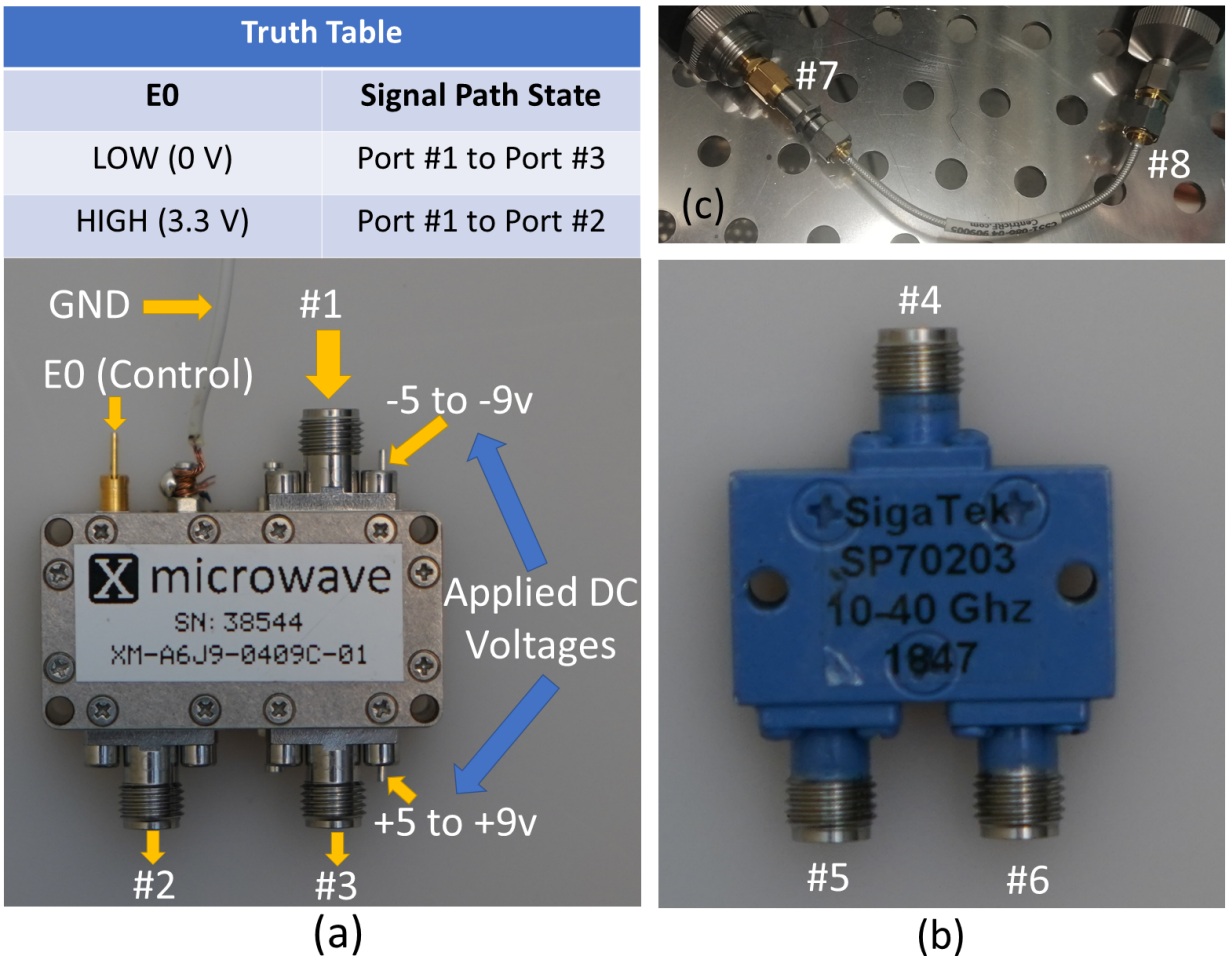
(**a**) PE42525 switch module. (**b**) SigaTek SP70203 power divider. (**c**) 4-inch formable cables from Centric RF.

A detailed view of the switch module, power divider, and 4-inch formable cable is shown in Figure 10. The switch module needs two input DC voltages that can be applied to the DC pins of the module. The whole module outer case is ground, and it is connected to one ground cable as shown in Figure 10a. The working principle of the switch module is explained by the truth table shown in the figure. The input power cable will be connected to port 1 and then according to the voltage level applied to E0 (Control) pin, the input signal will be switched either to port 2 or to port 3. When 0 V is applied to E0, the input signal will switch from port 1 to port 3, and port 2 will be the isolated port. When 3.3 V is applied to E0, the input signal switch to port 2 and port 3 will be the isolated port.

The voltage control board of the current switch module does not support the power division mode of the input signal at port 1 between port 2 and 3, even though in the datasheet of the switch chip [29] it is mentioned in state four (4) of the truth table for PE42525 chip that when both voltages (V1 and V2) are +3.3 V each, outputs (RF1 and RF2) of the switch chip are both ON. State 4 is not supported in the used x-microwave switch module, so only switching between port 2 and 3 is used for beam measurements. A 50 GHz network analyzer Agilent PNA 5245A is used to perform the S-parameters measurements for all components shown in Figure 10 in order to verify their performance and to record their insertion losses.

**Figure 11:**
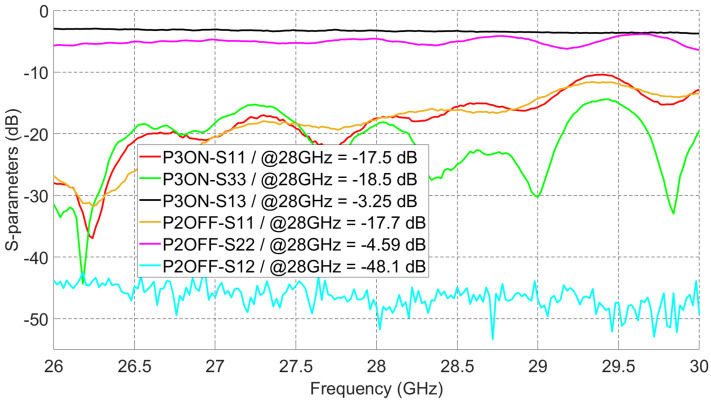
Measured S-parameters of the switch module.

Measured S-parameters of the switch module alone are shown in Figure 11. When 0 V is applied to E0, the input signal at port 1 passes to port 3 (P3ON-S13) with 3.25 dB insertion loss at 28 GHz. As reported in the datasheet of the switch chip, the chip alone has around 2 dB insertion loss, and the extra insertion loss is coming from connectors of the switch module and the boards inside it. At this switching state, the reflection coefficients of port 1 and 3 are less than −10 dB from 26 GHz to 30 GHz. To measure the isolation at port 2, the second VNA port is connected to switch module port 2, keeping 0 V applied at E0. As shown in the measured S-parameters, the isolation at port 2 (P2OFF-S12) is better than 40 dB for the whole frequency band. The reflection coefficient at port 1 (P2OFF-S11) is still less than −10 dB for the whole band; however, the reflection coefficient at port 2 (P2OFF-S22) is high (≈−5 dB), and this is expected because port 2 should be isolated.

**Figure 12:**
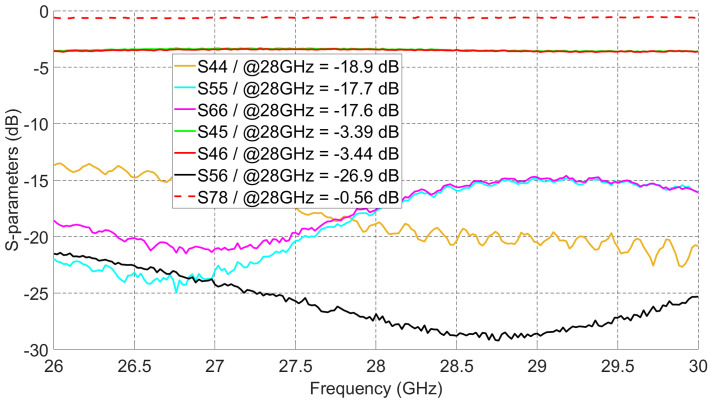
Measured S-parameters of the power divider and the formable cable.

In Figure 12, measured S-parameters of the power divider and the formable cable are shown. Within the targeted 5G frequency band, the power divider has a measured 3.4 dB power division ($S_{45}$ and $S_{46}$), by which 0.4 dB is coming from power divider insertion loss. All reflection coefficients ($S_{44}$, $S_{55}$, and $S_{66}$) are less than −10 dB. The isolation between ports 5 and 6 ($S_{56}$) is better than 20 dB from 26 GHz to 30 GHz. The measured insertion loss of the cable ($S_{78}$) is around 0.56 dB. The insertion losses of all feed network components will be added to the measured gain values of the switched beams to get the real radiation patterns’ gain of the proposed antenna shown in Figure 4.

### 4.3. Measured Radiation Patterns

**Figure 13:**
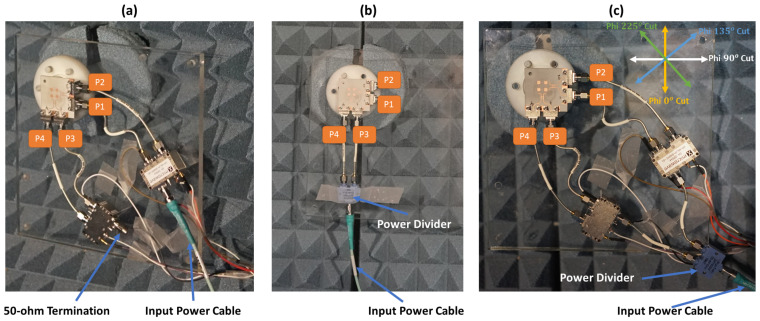
Radiation patterns measurement setup. (**a**) Measuring setup for beams 1, 3, 4, and 6. (**b**) Measuring setup for beams 2 and 5 (**c**). Measuring setup for beams 7, 8, 9, and 10.

The beam-switching capability of the proposed antenna system has been confirmed by measuring all ten (10) switched beams. Antenna measurement was done at Sabanci University Anechoic Chamber, where a standard horn antenna working from 26.5 GHz to 40 GHz was used for gain comparison and calculations. The setup for measuring all the switched beams is shown in Figure 13.

**Figure 14:**
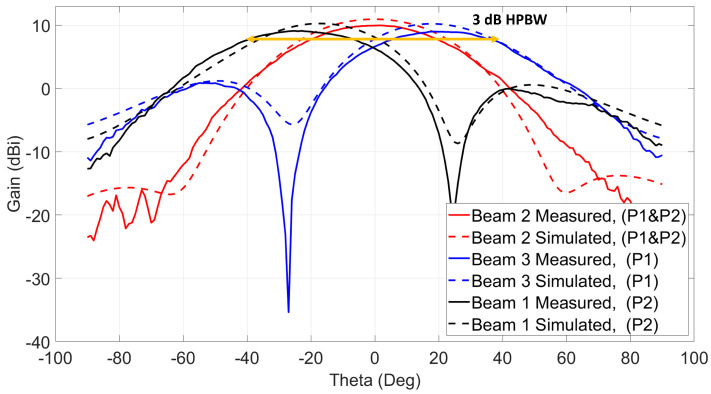
Three switched beams at 28 GHz using ports 1 and 2 / Phi = 0${}^{\circ }$ cut.

Beams 1, 3, 4, and 6 are measured by using the setup shown in Figure 13a. To obtain those beams, excitation from only one side of the antenna system is needed. To generate beams 1 and 3, ports 1 and 2 of the antenna shown in Figure 13a need to be excited, respectively. Similarly, beams 4 and 6 are excited using ports 3 and 4. As shown in the figure, at each side of the antenna, a Peregrine PE42525 SPDT switch module is connected to make the switching mechanism easier, and the input power cable is connected directly to the switch module. The switching mechanism of each switch module is controlled through voltage cables that apply the voltages mentioned in the truth table of Figure 10a. When one of the switch modules is used, for instance, while generating beams 1 and 3, the input of the other switch module is terminated with 50 ohms load. The measured radiation patterns for beams 1 and 3 across cut Phi 0${}^{\circ }$ are shown in Figure 14.

**Figure 15:**
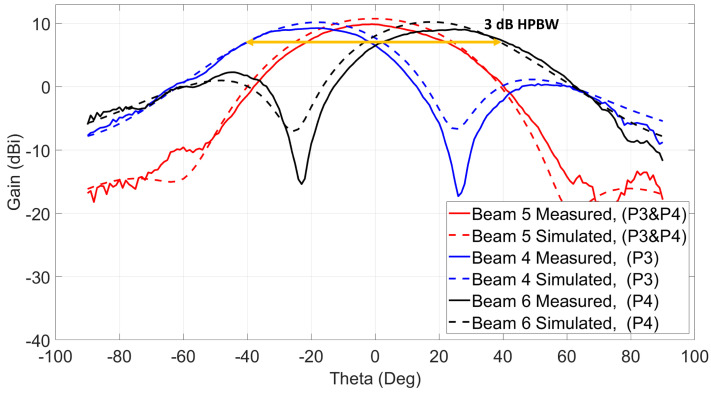
Three switched beams at 28 GHz using ports 3 and 4/Phi = 90${}^{\circ }$ cut.

Beam 1 is shifted to θ = −26${}^{\circ }$ with maximum measured gain equal to 9.1 dBi, and beam 3 is shifted to the other side and has a maximum gain of 8.9 dBi at θ = 26${}^{\circ }$. The measured losses shown before for the switch modules and the centric RF coaxial cables are de-embedded from the measured gains to know the real radiation patterns’ gain of the antenna system under test. Similarly, the second switch is used to excite ports 3 and 4 to generate beams 4 and 6. Those beams are measured across cut Phi 90${}^{\circ }$ with 9.1 dBi maximum measured gain at θ = −25${}^{\circ }$ for beam 4 and 9.1 dBi gain at θ = 25${}^{\circ }$ for beam 6 as shown in Figure 15. The simulated radiation patterns are plotted on top of the measured patterns, and they show a good agreement with at most 1 dBi difference between them.

Beams 2 and 5 are measured using the setup shown in Figure 13b. To generate those beams, the two ports from each side of the antenna system need to be excited at the same time. Since the switch module is not capable of doing power division between its outputs, a power divider is used to excite each beam. When ports 1 and 2 are excited simultaneously, as shown in the figure, beam 2 is generated at θ = 0${}^{\circ }$ with polarization across Phi = 90${}^{\circ }$ cut. Beam 5 is excited similarly by connecting the power divider to ports 3 and 4. This beam has a similar θ = 0${}^{\circ }$, but with polarization across Phi = 0${}^{\circ }$. The measured maximum gains for beams 2 and 5 are 9.9 dBi and 9.8 dBi, respectively, as shown in Figure 14 for beam 2 and Figure 15 for beam 5. The first three beams (1, 2, and 3) show a wide 3-dB HPBW from −40${}^{\circ }$ to +40${}^{\circ }$ across cut Phi = 0${}^{\circ }$, and a similar 3-dB HPBW is obtained for beams 4, 5, and 6 across cut Phi = 90${}^{\circ }$.

**Figure 16:**
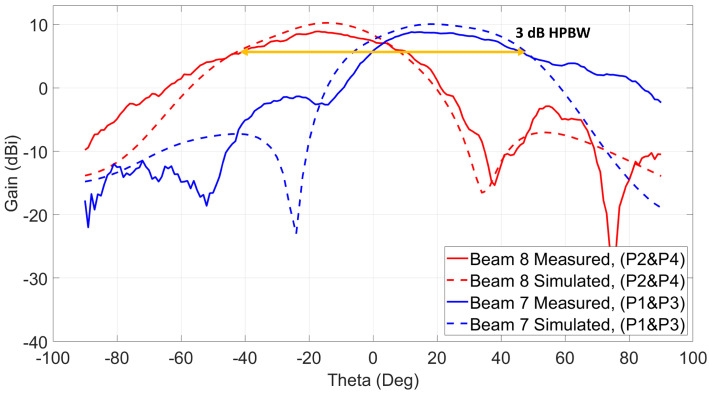
Two switched beams at 28 GHz using ports (2 and 4) and ports (1 and 3)/Phi = 225${}^{\circ }$ cut.

**Figure 17:**
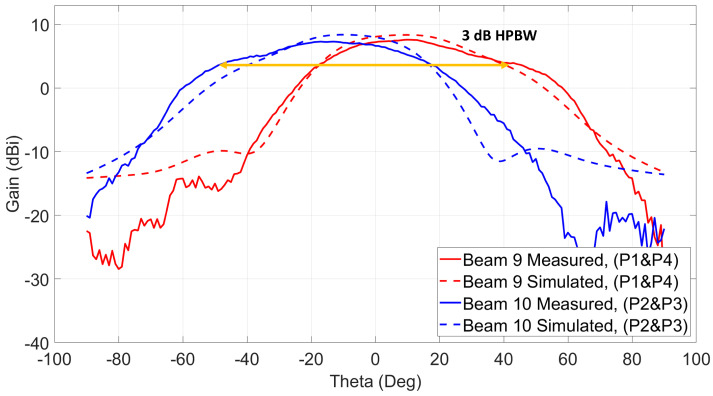
Two switched beams at 28 GHz using ports (1 and 4) and ports (2 and 3)/Phi = 135${}^{\circ }$ cut.

The measurement setup shown in Figure 13c is used to measure beams 7, 8, 9, and 10. Since those cross beams are excited using a simultaneous two-port excitation from both sides of the antenna system, both switch modules are used at the same time to excite each of the beams. As shown, a power divider is used to connect both inputs of the switches, and the input power cable is connected to the input of the power divider. To generate the seventh beam, ports 1 and 3 need to be excited at the same time, and the eighth beam is generated when ports 2 and 4 are excited. Both beams are across Phi = 225${}^{\circ }$. The measured patterns are shown in Figure 16. The maximum measured gain for beam 7 is 8.8 dBi at θ = 17${}^{\circ }$, and beam 8 shows a maximum gain of 8.9 dBi at θ = −17${}^{\circ }$. The measured 3-dB HPBW of both beams is around 80${}^{\circ }$. The last two beams (9 and 10) are excited similarly, and their radiation patterns across Phi = 135${}^{\circ }$ are shown in Figure 17. Those beams show around 90${}^{\circ }$ 3-dB HPBW, which is wider than the previous reported ones.

In Table 2, all measured results together with the corresponding port excitations are reported. As shown, the average measured gain for the 10 switched beams is around 8.9 dBi. The 10 switched beams cover the four quadrants of the 2D space.

**Figure 18:**
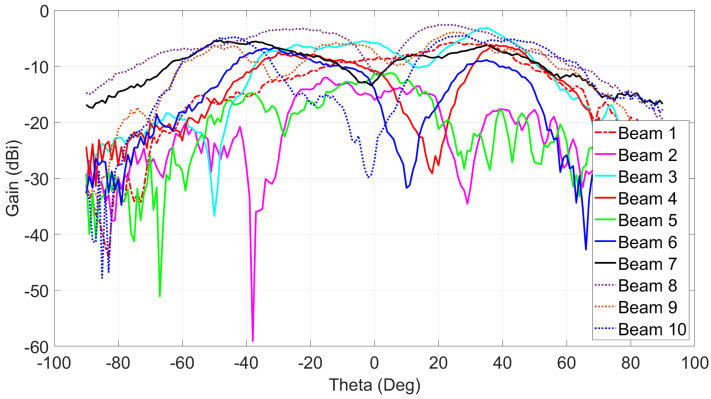
Measured cross-polarization gains for the ten (10) switched beams at 28 GHz.

**Figure 19:**
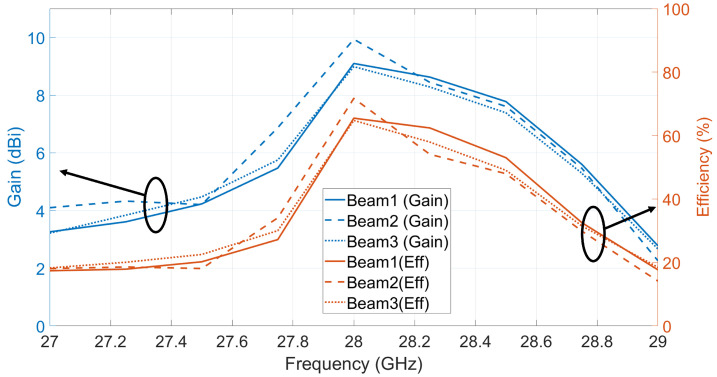
Measured gain vs. frequency and calculated efficiency for beams 1, 2, and 3.

Figure 18 shows the measured cross-polarization levels for the ten (10) switched beams. Taking into account 90${}^{\circ }$ 3-dB HPBW and the average measured gain for all beams as 8.9 dBi, the maximum cross-polarization level is −5 dBi and the minimum cross-polarization level is −30 dBi, and these will correspond to the co/cross-polarization ratio of 14 dB and 39 dB, respectively. These low cross-polarization levels are due to the high inter-port isolations (shown in Figure 6) between the dual-polarized ports. In order to investigate the performance of the radiation patterns vs. frequency, the maximum measured gains for beams 1, 2, and 3 are recorded at multiple frequencies from 27 GHz to 29 GHz as shown in Figure 19. Add to that, the efficiency for each beam vs. frequency is calculated. The efficiency is calculated using simulated directivity values of those beams at each frequency with measured realized gain at each frequency. As shown in the figure, the measured gains and calculated efficiencies for those beams reaches maximum at 28 GHz and drops when moving away from the resonance frequency (28 GHz) of the 2 × 2 series fed antenna array. This is caused by the narrow radiation bandwidth of the regular microstrip patch antenna that is used as an array element to build the antenna array. Despite the fact that the antenna system reached wide 10 dB impedance bandwidth (as shown in Figure 5), the antenna gain vs. frequency is limited by the narrow band microstrip patch antenna. The other seven (7) switched beams will behave in a similar way vs. frequency.

### 4.4. Comparison and Discussion

In Table 3, a comparison is carried out between this work and different antenna arrays reported in the literature. A single-layer dual-polarized 2 × 2 antenna array operating at 28 GHz with five switched beams is proposed in [4]; however, the antenna uses phase shifters to steer the antenna beam, and those phase shifters have losses that can reach 6 dB. In [18], a multi-layer design is proposed by which Rotman lens is used as a beamforming network, and it feeds the 7 × 4 antenna array through slots; however, the antenna generates five beams with a single polarization. In [21,23,24,25], multi-layer antenna topologies operating at 28 GHz and 38 GHz are proposed. Antennas in those works use SIW butler matrixes as a beamforming network which has higher complexity than the beamforming network of this work. In work [23], a dual-polarized 2 × 2 multi-layer antenna array is proposed which can do 2D beam-scanning using eight ports and SIW beamforming network to generate eight beams having an average measured gain of 10 dBi; however, in this work, only a four-port single-layer antenna with beamforming network could generate 10 switched beams with 8.9 dBi measured gain. In works [21,24], larger array sizes are used to achieve higher measured gains, but they have 1D scanning capability and single polarization. The work mentioned in [25] proposes a magneto-electric dipole array with circular polarization. Even though some of the mentioned works have high antenna beam gains and wide-band radiation patterns, they have high fabrication complexity, and some of the arrays are large and expensive for mobile phone devices. When comparing the previous works with the dual-polarized antenna system proposed in this paper, it is seen that the proposed antenna has a very simple and low-controlled beamforming network which is composed of two 3-dB couplers. The antenna uses only four ports to generate up to ten (10) switched beams with an average measured gain of 8.9 dBi, and it has maximum steering angle of ±26${}^{\circ }$ with 3-dB coverage from 80${}^{\circ }$ to 90${}^{\circ }$ in 2D space. Additionally, the antenna is a low-cost single-layer design with a simple fabrication process, and it has a compact size (18 × 18 mm${}^{2}$) that suits regular-sized 5G mobile phone devices. The antenna has 3-dB coverage from 80${}^{\circ }$ to 90${}^{\circ }$ in 2D space, and it has a maximum of ±26${}^{\circ }$ beam-steering angle.

## 5. Conclusions

A 28 GHz 2 × 2 dual-polarized four-port single-layer antenna system is introduced. The antenna uses a very simple network composed of two 3 dB couplers to generate 10 switched beams in 2D space. The antenna design is implemented on 0.203 mm thick low-loss (tanδ = 0.0027) Rogers 4003C substrate, and it has a compact size of 37.2 × 37.2 mm${}^{2}$ including the ground plane. The size of the antenna without connectors is 18 × 18 mm${}^{2}$. The measured impedance bandwidth of the antenna reaches 4 GHz (14.3%, from 26 GHz to 30 GHz) for all ports. Measured peak isolation between any dual-polarized ports of the antenna is better than 30 dB. All radiation patterns have been measured and verified with a low-controlled measurement setup. The antenna has an average measured realized gain of 8.9 dBi and around 10 dB side lobe level (SLL) for all beams. The antenna has 3 dB coverage from 80${}^{\circ }$ to 90${}^{\circ }$ in 2D space, and it has a maximum of ±26${}^{\circ }$ beam-steering angle. The presented antenna is a good candidate for 5G mobile devices and high data rate applications that need antenna designs with low cost, low complexity, and compact size.

## Figures and Tables

**Table 1:** Antenna port excitations to generate ten (10) switched beams.

Coupler 1		Coupler 2		Beam No.
P1	P2	P3	P4	
OFF	ON	OFF	OFF	Beam 1
ON	ON	OFF	OFF	Beam 2
ON	OFF	OFF	OFF	Beam 3
OFF	OFF	ON	OFF	Beam 4
OFF	OFF	ON	ON	Beam 5
OFF	OFF	OFF	ON	Beam 6
ON	OFF	ON	OFF	Beam 7
OFF	ON	OFF	ON	Beam 8
ON	OFF	OFF	ON	Beam 9
OFF	ON	ON	OFF	Beam 10

**Table 2:** Maximum measured gains of the ten (10) switched beams.

Beam No.	Excited Port/s	Theta (θ)	Phi (ϕ)	Measured Gain (dBi)
1	P2	−26${}^{\circ }$	0${}^{\circ }$	9.1
2	P1 and P2	0${}^{\circ }$	0${}^{\circ }$	9.9
3	P1	26${}^{\circ }$	0${}^{\circ }$	8.9
4	P3	−25${}^{\circ }$	90${}^{\circ }$	9.1
5	P3 and P4	0${}^{\circ }$	90${}^{\circ }$	9.8
6	P4	25${}^{\circ }$	90${}^{\circ }$	9.1
7	P1 and P3	17${}^{\circ }$	225${}^{\circ }$	8.8
8	P2 and P4	−17${}^{\circ }$	225${}^{\circ }$	8.9
9	P1 and P4	17${}^{\circ }$	135${}^{\circ }$	7.6
10	P2 and P3	−17${}^{\circ }$	135${}^{\circ }$	7.1

**Table 3:** Comparison of the proposed antenna performance with other works from the literature.

Ref.	Layer Technology	Antenna and Topology	Frequency (GHz)	Beam Coverage Angle	Number of Beams	Average Measured Gain (dBi)	Polarization
[4]	Single Layer	Phase Shifters + 2 × 2 Array	28	±24${}^{\circ }$	5	12	Dual
[18]	Multi-Layer Slot	Rotman Lens + 7 × 4 Array	24	±30${}^{\circ }$	5	/	Single
[21]	Multi-Layer SIW	Butler Matrix + 8 × 10 Array	38	±36${}^{\circ }$	4	19.8–21	Single
[23]	Multi-Layer SIW	Butler Matrix + 2 × 2 Array	28	±30${}^{\circ }$	8	10	Dual
[24]	Multi-Layer SIW	Butler Matrix + 2 × 7 Array	28	±45${}^{\circ }$	5	13.2	Single
[25]	Multi-Layer SIW	Butler Matrix + ME Dipole Array	28	±40${}^{\circ }$	5	12.5	Circular
This work	Single Layer	Simple Feed + 2 × 2 Array	28	±26${}^{\circ }$	10	8.9	Dual
